# Latent Trajectories and Regional Differences in Carbon Monoxide Mortality Across Provinces of Iran

**DOI:** 10.34172/aim.34565

**Published:** 2025-08-01

**Authors:** Farzad Maleki, Zahedeh Khoshnazar, Azadeh Noohi, Mohammad Reza Taherian, Fatemeh Majdolashrafi, Seyed Saeed Hashemi Nazari

**Affiliations:** ^1^Department of Epidemiology, School of Public Health & Safety, Shahid Beheshti University of Medical Sciences, Tehran, Iran; ^2^Prevention of Cardiovascular Disease Research Center, Shahid Beheshti University of Medical Sciences, Tehran, Iran

**Keywords:** Carbon monoxide poisoning, Cluster, Growth mixture model, Iran, Mortality rate

## Abstract

**Background:**

Considering the geographic and socio-economic heterogeneity across Iranian provinces, studying carbon monoxide poisoning (COP) mortality trends can provide insight for decision-making and necessary interventions. This study aimed to model the trend of COP mortality across 31 provinces of Iran from 2011 to 2022.

**Methods:**

The current study used data from the Iranian Legal Medicine Organization (ILMO), the official body responsible for certifying and registering all suspected COP deaths in Iran, from 2011 to 2022. The annual and 10-year cumulative mortality rates were calculated by sex for all provinces. The growth mixture model (GMM) was employed to classify provinces according to the magnitude of alterations in the COP mortality rate concerning the intercept and slope parameters, utilizing the R software and the lcmm package.

**Results:**

From 2011 to 2022, 9555 deaths due to COP were reported. The national 10-year cumulative mortality rate was 10.04 (95% CI: 8.34–11.75) per 100,000 for both sexes, 14.74 (12.37–17.12) for males, and 5.02 (4.06–6.34) for females. The Alborz Province for both sexes and males and the East Azerbaijan Province for females reported the highest mortality over ten years: 18.69 (17.25–20.19), 26.21 (23.93- 28.6), and 11.13 (7.34- 9.88) per 100,000 persons, respectively. The GMM results indicated that the overall COP mortality rate in class 2 and class 4 increased approximately by three and two times, respectively.

**Conclusion:**

The rising trend of COP mortality in several provinces requires urgent interventions, focusing on safety and modern heating. Public awareness and CO detector installation, especially in colder regions, are crucial for preventing CO poisoning.

## Introduction

 Carbon monoxide kills thousands of people worldwide every year,^1^ and it is one of the most important causes of disability, severe complications, and mortality in the world.^2^ According to Global Burden of Disease (GBD) estimates, in 2021, the global mortality rate of unintentional carbon monoxide poisoning (COP) was 3.66 per 100,000 persons, yielding 18.1 million years of life lost across all ages.^3^ The overall annual incidence of COP in the Middle East and North Africa is 13.37 cases per 100,000 persons.^4^ The incidence of COP in Iran is estimated to be 38.91 per 100,000 persons.^5^ Based on reports between 1990 and 2021, prevalence was higher in females, while deaths were reported more frequently in males.^6^ A recent systematic review in Iran indicated that 40.12% of COP cases were in men and 59.88% were in women.^5^ In contrast, another study in Iran revealed that 75% of the cases were male.^7^ The global trend of the COP mortality rate has been decreasing from 2000 to the present.^8,9^ Also, studies in Iran have shown a decreasing trend in the past two decades.^7,10^ Another study in southern Iran from 2004-2019 showed that the COP mortality rate is stable, with an insignificant decreasing trend.^11^

 CO is an odorless and colorless gas with very low irritation.^3,9^ This gas is produced from the incomplete burning of fuels such as wood, coal, gasoline, and natural gas, and its primary sources are gas stoves, boilers, fires, portable generators, gas heaters, fossil fuel burning devices, and clogs.^3^ Non-occupational COP is notably higher in residential settings, particularly during cold seasons when windows are closed and heating appliances are utilized.^12^ Common symptoms of COP include headache, nausea and vomiting, dizziness, general weakness, and a change in mental status.^13^ To decrease the complications caused by COP, immediately removing the source of poisoning and performing hyperbaric oxygen therapy is recommended, but there is still no natural treatment for poisoning with this gas, which emphasizes the importance of prevention.^14^ Exposure to CO presents a significant health risk, as individuals can experience severe or fatal poisoning without realizing that they have been exposed.^15^

 Economic and geographic factors influence individuals’ vulnerability to COP.^16^ Generally, as the sociodemographic index (SDI) scores increase, the annual percentage change values decrease. Therefore, higher SDI values and per capita income may reduce COP fatalities.^17,18^ The mortality rates for COP in Central and Eastern Sub-Saharan Africa, Southern Latin America, and high-income North America have been increasing. At the same time, the remaining 16 regions, including tropical Latin America, Europe, and the Caribbean, have experienced a decline in mortality rates.^9^ Furthermore, the primary cause of CO poisoning is using non-standard traditional heating systems, which are more common in countries with low socioeconomic status.^19^ The recent meta-analysis study conducted in Iran also concluded that CO exposure is linked to socioeconomic status, educational levels, and healthcare quality.^5^

 Iran’s provinces are characterized by significant geographic and socioeconomic heterogeneity,^20^ which may drive divergent trends in COP mortality. While earlier studies have described general patterns, none have employed the GMM —a method uniquely suited to identify non-linear trajectories and cluster provinces with distinct mortality trends. GMM’s ability to uncover latent subgroups enables policymakers to move beyond one-size-fits-all strategies, as provinces within the same cluster likely share modifiable risk factors (e.g., heating infrastructure, socioeconomic conditions). This approach is particularly critical in Iran, where tailored interventions are urgently needed to address regional disparities. Additionally, this study provides the most recent available data (2011–2022), filling a gap in timely evidence for decision-making. By incorporating hotspot analysis, the methodology identifies priority regions requiring targeted resource allocation, ensuring prevention strategies align with geographic urgency. Collectively, these advancements address a critical methodological and practical void, equipping stakeholders with tools to design context-specific interventions and reduce preventable deaths. Therefore, the present study uses growth mixture modeling and hotspot analysis to model latent trajectories and regional disparities in carbon monoxide mortality across the provinces of Iran.

## Materials and Methods

 In this study, COP mortality data from March 21, 2011, to March 21, 2022, were extracted from the Iranian Legal Medicine Organization (ILMO). At the end of each year, the aggregated COP statistics are published on the ILMO website and made publicly available.

 In Iran, confirmation of the diagnosis of suspicious deaths is done by trained and specialized LMO physicians in the prepared standard death certificate forms. Based on the standard death certificates, the expert physicians of the autopsy centers of each province collect the data and subsequently send it to the central office. According to Iranian law, all cases of suspicious deaths must be referred to the ILMO to evaluate and determine the leading cause of death. Deaths caused by COP are also one of the criteria for a suspicious death. Therefore, the LMO database is the most comprehensive source of COP death reports in Iran. Furthermore, confirmation methods follow uniform guidelines across all provinces, ensuring consistency in the diagnosis process. These cases are categorized according to the International Classification of Diseases, Tenth Revision (ICD-10 codes: X47.0 to X47.9).

 For mortality rate calculations, in instances where population data for specific years were unavailable, the following approach was adopted: the 2011 census results were utilized to approximate populations for the years 2012 through 2015; the 2016 census results were employed to estimate populations for the years 2017 to 2021; the 2022 census findings were applied to calculate population for 2023. The average annual growth rates for the country and its provinces were also used in the estimations. The 10-year mortality rate was calculated by dividing the deaths within ten years by the average number of at-risk people. Annual and cumulative mortality rates were calculated with 95% confidence intervals for all provinces and sexes.

###  Growth Mixture Model (GMM)

 We used GMM, considering the probable heterogeneity of mortality rate changes and to identify homogeneous subgroup trends over time. In GMM, provinces are classified based on the changing rate of the desired variable over time. The scalar form of a *K*-Class GMM with a random intercept and slope and class-specific error variance is defined by the following formula:


(1) $$y_{it}^{k}=\beta_{0}^{k}+b_{0i}^{k}+\left(\beta_{1}^{k}+b_{1i}^{k}\right)\times t+\varepsilon_{it}^{k}$$

Assumptions: $\begin{array}{r} b_{ji}^{k}∼N\left(0,\sigma_{b_{j}^{k}}^{2}\right),j=0,1,2\\ \varepsilon_{it}^{k}∼N\left(0,\sigma_{\varepsilon_{kt}}^{2}\right)\\ \text{cov}\left(b_{ji}^{k},b_{hi}^{k}\right)\neq 0,j\neq h,h=0,1,2 \end{array}$

 Where *t*= 1,…,*T* denotes time (year), *i* = 1,…,*N* denotes province, and $y_{it}$ denotes the COP mortality rate during the year *t* in province *i.k* = 1,…,*K* denotes cluster or latent class.^21^ $\beta_{0}^{k}$ and $\beta_{1}^{k}$ represent fixed effects for cluster *k*, the class-specific intercept (the rate at the start of the study period) and slope (linear trend over the study period); $b_{0i}^{k}$ and $b_{1i}^{k}$ represent random effects that capture province differences in class *k* (inter-province variability); and $\varepsilon_{it}^{k}$ represents errors (intra-province variability) and is class-specific. In other words, each class can have a different average COP mortality rate at the start and different slopes over the study period. Provinces can deviate from the class averages (intercept and slope) by various degrees, and the magnitude of provinces’ deviation from the class average can differ across classes. The statistical significance of the intercept, slope, and quadratic terms was assessed using Wald tests within the GMM framework; p-values below 0.05 indicated significant trend changes.^22^ The models were built using the lcmm package in the R software, version 4.3.2.^23^

###  Goodness of Fit and Assessment of Model Adequacy

 Different criteria determine the optimal number of classes that form the most distinctive classes. We tested models ranging from one to seven classes to identify the optimal number of latent trajectories. The optimal model was selected by comparing the Akaike information criterion (AIC), Bayesian information criterion (BIC), and Sample-Size adjusted (ssBIC),^22^ and lower values of these information criteria indicate the optimal number of classes. Entropy statistics were used to evaluate the quality of the classification, and a value of 0.8 or higher was considered a good classification.^21^ Both linear and quadratic terms were tested to capture potential non-linear trends. No additional covariates were included beyond time and province classification. Model selection results are presented in Table S1. To account for possible heterogeneity by sex, separate GMMs were fitted for males, females, and the total population rather than including an interaction term within the model.

###  Hotspot Identification

 We used hotspot analysis to identify clusters of COP mortality rates over different years. These hotspots indicate areas with a high concentration of the events being studied. First, Moran’s index was used to measure spatial autocorrelation based on feature locations and feature values. The results of this evaluation indicate the distribution of the expressed pattern. Its value ranges from -1 to + 1, where -1 represents perfect dispersion, meaning that similar values are evenly spread out across space, 0 indicates no spatial correlation, and + 1 represents perfect clustering, meaning that similar values are highly concentrated in specific areas.^24^ The formula for Moran’s Index(*I*) is expressed as follows:


$$I=\frac{n}{W}.\frac{\sum_{i=1}^{n}\sum_{j=1}^{n}w_{ij}\left(x_{i}-\bar{x}\right)\left(x_{j}-\bar{x}\right)}{\sum_{i=1}^{n}{\left(x_{i}-\bar{x}\right)}^{2}}$$

Where: *n* is the number of spatial units, *W* is the sum of all weights, *w*_ij_ represents the spatial weight between units *i* and j, *x*_i_ and *x*_j_ are the values at locations *i* and *j*, and $\bar{x}$ is the mean of the values. This formula quantifies how similar or dissimilar values are across space, helping to identify patterns of clustering or dispersion.

 Getis-Ord Gi statistics were used afterwards to recognize COP hotspots, where a higher index score and a lower p-value indicate a clustering of the events.^25^ The formula for the Getis-Ord Gi* statistic is expressed as follows:


$$G_{i}^{*}=\frac{\sum_{j=1}^{n}w_{ij}x_{j}-\bar{x}\sum_{j=1}^{n}w_{ij}}{S\sqrt{\frac{\left(n\sum_{j=1}^{n}w_{ij}^{2}\right)-{\left(\sum_{j=1}^{n}w_{ij}\right)}^{2}}{\left(n-1\right)}}}$$

Where: $G_{i}^{*}$ is the Getis-Ord statistic for the feature *i*, *n* is the total number of features, *w*_ij_ is the spatial weight between feature *i* and feature *j*, *x*_j_ is the value of feature *j*, $\bar{x}$ is the mean of all values, and *S* is the standard deviation of the values. Significance of spatial clusters was assessed using Z-scores, with |Z| > 1.96 indicating *P* < 0.05.

## Results

 From 2011 to 2022, 9555 deaths were reported due to COP. Seven thousand eight deaths were male (74.39%), and 2447 were female (25.61%). The crude mortality rate of COP changed from 1.28 in 2011 to 1.08 in 2022 per 100,000 population. The overall male-to-female ratio was 2.87 (Table 1).

**Table 1:** Overall Annual Mortality Rate of Carbon Monoxide Poisoning by Sex in Iran, 2011–2022

**Year**	**Number of deaths**			**Mortality rate (per 100,000)**			**Male/female ratio**
	**Female**	**Male**	**Both sexes**	**Female**	**Male**	**Both sexes**	
2011	253	712	965	0.68	1.88	1.28	2.76
2012	207	490	697	0.55	1.28	0.92	2.33
2013	240	588	828	0.63	1.51	1.08	2.40
2014	158	468	626	0.41	1.19	0.80	2.90
2015	144	482	626	0.37	1.21	0.79	3.27
2016	216	620	836	0.55	1.53	1.05	2.78
2017	202	564	766	0.50	1.37	0.94	2.74
2018	176	573	749	0.43	1.38	0.91	3.21
2019	194	590	784	0.47	1.41	0.95	2.97
2020	235	691	926	0.57	1.64	1.11	2.88
2021	208	631	839	0.50	1.49	1.00	2.97
2022	214	699	913	0.51	1.63	1.08	3.20

 The 10-year cumulative COP mortality rate in the Alborz Province was 18.69 (17.25–20.19) for both sexes and 26.21 (23.93–28.60) for males, which was approximately two times greater than the national average of 10.04 (8.34–11.75) for both sexes and 14.74 (12.37–17.12) for males. For females, 11.13 (7.34–9.88) in the East Azerbaijan province was approximately two times greater than the national average of 5.02 (4.06–6.34). Furthermore, the lowest mortality rate was assigned to the Hormozgan Province for both sexes [1.1 (0.67–1.69)] and males [1.83 (1.07–2.91)], and the Yazd Province for females [5.02 (1.88–4.93)], which were one-tenth of the national average (Table 2).

**Table 2:** Ten-Year Cumulative Mortality Rates of Carbon Monoxide Poisoning for Males, Females, and Total per 100,000 Population (Sorted by Mortality Rate)

**ID**	**Province**	**Overall rate (95% CI)**	**Province**	**Male rate (95% CI)**	**Province**	**Female rate (95% CI)**
1	Alborz	18.69 (17.25- 20.19)	Alborz	26.21 (23.93- 28.6)	Eastern Azerbaijan	11.13 (7.34- 9.88)
2	Zanjan	17.45 (15.22- 19.85)	Northern Khorasan	24.97 (20.95- 29.28)	Western Azerbaijan	10.97 (1.34- 2.75)
3	North Khorasan	16.57 (14.16- 19.21)	Semnan	24.69 (20.32- 29.41)	Ardabil	10.7 (8.39- 13.39)
4	Semnan	16.57 (13.93- 19.5)	Zanjan	23.66 (20.14- 27.47)	Esfahan	8.87 (3.58- 5.22)
5	Ardabil	15.49 (13.54- 17.58)	Markazi	21.2 (18.29- 24.35)	Alborz	8.71 (9.36- 12.75)
6	Markazi	15.13 (13.32- 17.08)	Qazvin	21.15 (18.09- 24.48)	Ilam	8.55 (4.03- 10.15)
7	Qazvin	15.05 (13.14- 17.12)	Ardabil	20.07 (17.05- 23.34)	Bushehr	8.2 (0.41- 2.35)
8	Eastern Azerbaijan	13.71 (12.65- 14.82)	Esfahan	19.31 (17.82- 20.88)	Tehran	8.11 (5.48- 6.64)
9	Tehran	12.21 (11.66- 12.77)	Eastern Azerbaijan	18.71 (17.02- 20.48)	Charmahal and Bakhtiari	8.1 (2.11- 5.71)
10	Esfahan	11.93 (11.06- 12.84)	Tehran	18.31 (17.39- 19.25)	Southern Khorasan	6.62 (2.04- 5.68)
11	Ilam	11.64 (9.16- 14.53)	Kohkiloyeh and Boyerahmad	17.62 (13.85- 21.95)	Khorasan Razavi	6.57 (4.10- 5.59)
12	Hamedan	11.49 (10.03- 13.07)	Ilam	16.5 (12.46- 21.22)	North Khorasan	6.38 (5.71- 11.09)
13	Lorestan	11.29 (9.85- 12.85)	Charmahal and Bakhtiari	16.46 (13.27- 20.06)	Khuzestan	6.04 (1.21- 2.29)
14	Charmahal and Bakhtiari	10.12 (8.29- 12.21)	Hamedan	16.32 (13.93- 18.91)	Zanjan	4.81 (8.58- 14.12)
15	Kermanshah	10.12 (8.82- 11.54)	Fars	15.36 (13.96- 16.84)	Semnan	4.38 (5.55- 11.55)
16	Kohkiloye and Boyerahmad	10.11 (8.01- 12.54)	Kurdistan	14.63 (12.28- 17.24)	Sistan and Baluchistan	4.34 (0.76- 2.01)
17	Fars	9.79 (8.98- 10.66)	Lorestan	14.39 (12.16- 16.87)	Fars	4.08 (3.33- 4.95)
18	Kurdistan	9.57 (8.18- 11.12)	Kermanshah	13.78 (11.7- 16.08)	Qazvin	4.02 (6.62- 11.18)
19	Khorasan Razavi	9.11 (8.43- 9.83)	Khorasan Razavi	13.35 (12.21- 14.56)	Qom	3.6 (2.13- 5.08)
20	Mazandaran	8.47 (7.54- 9.47)	Yazd	13.24 (10.64- 16.21)	Kurdistan	3.54 (3.07- 6.03)
21	Yazd	8.33 (6.82- 10.11)	Southern Khorasan	12.92 (10.01- 16.33)	Kerman	3.38 (1.29- 2.71)
22	South Khorasan	8.3 (6.59- 10.27)	Qom	12.92 (10.49- 15.71)	Kermanshah	3.16 (4.92- 8.09)
23	Qom	8.24 (6.81- 9.85)	Mazandaran	12.87 (11.3- 14.58)	Kohkiloyeh and Boyerahmad	3.15 (1.16- 4.73)
24	Kerman	5.67 (4.89- 6.52)	Kerman	9.27 (7.91- 10.78)	Golestan	3.15 (2.15- 4.49)
25	Gilan	5.67 (4.81- 6.64)	Gilan	8.19 (6.74- 9.84)	Gilan	2.52 (2.26- 4.26)
26	Golestan	5.52 (4.54- 6.64)	Western Azerbaijan	8.15 (6.89- 9.56)	Lorestan	1.96 (6.38- 10.11)
27	Western Azerbaijan	5.1 (4.38- 5.92)	Golestan	7.87 (6.24- 9.77)	Mazandaran	1.91 (3.12- 5.08)
28	Bushehr	3.21 (2.28- 4.38)	Bushehr	5.06 (3.48- 7.06)	Markazi	1.69 (6.89- 11.21)
29	Khuzestan	2.85 (2.40- 3.36)	Sistan and Baluchestan	4.17 (3.19- 5.34)	Hormozgan	1.27 (0.07- 0.98)
30	Sistan and Baluchestan	2.74 (2.17- 3.42)	Khuzestan	3.98 (3.24- 4.84)	Hamedan	1.09 (5.01- 8.42)
31	Hormozgan	1.1 (0.67- 1.69)	Hormozgan	1.83 (1.07- 2.91)	Yazd	0.34 (1.88- 4.93)
	National	10.04 (8.34 – 11.75)	National	14.74 (12.37 – 17.12)	National	5.02 (4.06 – 6.34)

**Figure 1:**
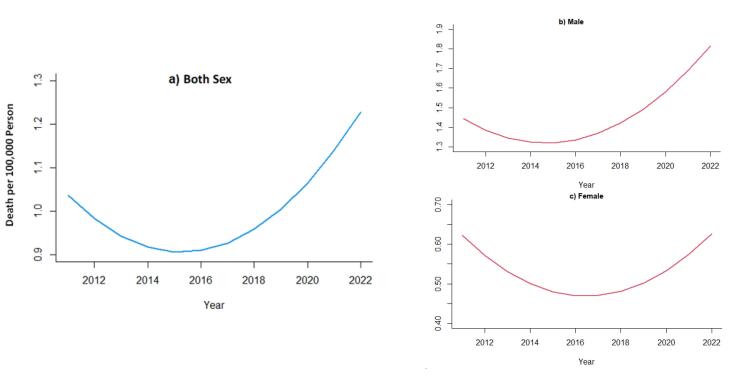
Overall Trends of COP Mortality Rates in Iran from 2011 to 2022 Using the GMM for Both Sexes, Males, and Females per 100,000 Persons

 The overall linear mortality rate increased by 0.017 every year, from 0.91 in 2011 to 1.11 in 2022 (Figure S1). The trend of COP mortality rates in Iran from 2011 to 2022, as estimated by the quadratic model, indicated that overall and male COP mortality rates have a J-shaped trend that declined from 2012 to 2016, followed by a steady increase until 2022. In contrast, the females showed a U-shaped trend (Figure 1).

 The trend of COP mortality rates was evaluated using a GMM with one trajectory. The average initial mortality rate (intercept) was 1.106 (SE = 0.109, *P* < 0.001), with a negative slope of -0.076 (SE = 0.023, *P* < 0.004) suggesting a decreasing trend initially, while the positive quadratic term of 0.007 (SE = 0.002, *P* < 0.001) indicates an upward turn in recent years. For males, the trend was more pronounced with a higher initial rate (intercept of 1.521, SE = 0.159, *P* < 0.001), a steeper decline (slope of -0.086, SE = 0.042, *P* < 0.041), and a significant upward turn indicated by the quadratic term of 0.009 (SE = 0.003, *P* < 0.003). The females had a lower initial rate (intercept of 0.684, SE = 0.078, *P* < 0.001) and a decline (slope of -.066, SE = 0.021, *P* < 0.001), with an increase later (quadratic term of 0.0052, SE = 0.001, *P* < 0.008) (Table S1).

 Seven models ranging from one to seven classes were developed to determine the optimal number of latent classes. Given the lower AIC, BIC, and ssBIC values and higher entropy collectively, the optimal model was observed in the fourth class for males and females and the fifth class for both sexes. The values of the entropy statistic for the selected models were calculated as 0.918, 0.86, and 0.937 for males, females, and both sexes, respectively (Table S2).

**Figure 2:**
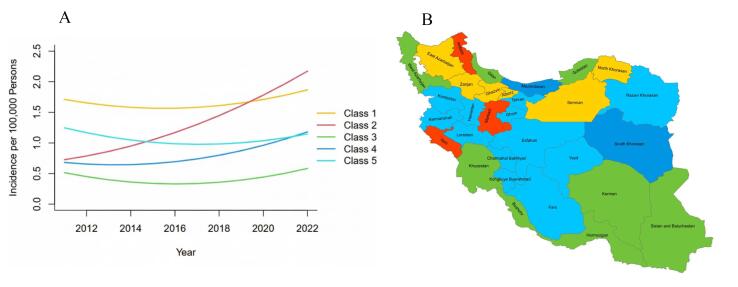
Overall Carbon Monoxide Death Rates Across Time in a 5-Cluster Growth Mixture Model (Panel A), Geographic Illustration of GMM Classification for Trends in Different Classes from 2011 to 2022 (Panel B)

 The GMM analysis revealed five distinct patterns in COP mortality trends across the Iranian provinces from 2011 to 2022. Among these patterns, Class 1 provinces (including Alborz, East Azarbaijan, North Khorasan, Qazvin, Semnan, Zanjan) stood out with the highest initial mortality rate of 1.78 per 100,000 population. While these provinces maintained relatively stable mortality levels with only a marginal annual increase (slope: + 0.01), their rates increased slightly from 1.71 to 1.86 per 100,000 over the study period. In striking contrast, Class 2 provinces (Ardabil, Ilam, and Markazi) presented a concerning pattern. Starting from the lowest baseline mortality of 0.68 per 100,000, these provinces experienced the most dramatic acceleration in COP deaths, with an annual increase of 0.13 per 100,000, nearly tripling their mortality rate from 0.72 to 2.15 over twelve years. This rapid escalation occurred despite their initially favorable position. The largest group of provinces, classified as Class 5 and including major regions like Tehran, Esfahan, and Fars, demonstrated a different trajectory. Beginning with a high baseline mortality of 1.34 per 100,000, these provinces showed modest annual improvement (slope: -0.01), with rates declining from 1.25 to 1.14 per 100,000. Meanwhile, Class 4 provinces (Mazandaran and Southern Khorasan) followed an intermediate path, starting at 0.72 per 100,000 and experiencing a moderate upward trend (slope: + 0.04), increasing their mortality to 1.18 per 100,000 by 2022 (Figure 2, Table S3, Table S4).

**Figure 3:**
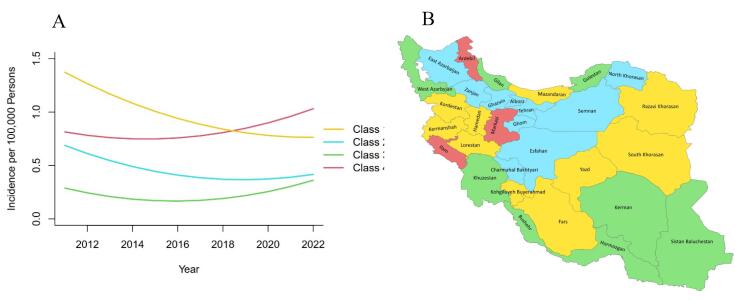
Female Carbon Monoxide Poisoning Mortality Rates Across Time in a 4-Cluster Growth Mixture Model (Panel A), Geographic Illustration of GMM Classification for Trends in Different Classes from 2011 to 2022 (Panel B)

**Figure 4:**
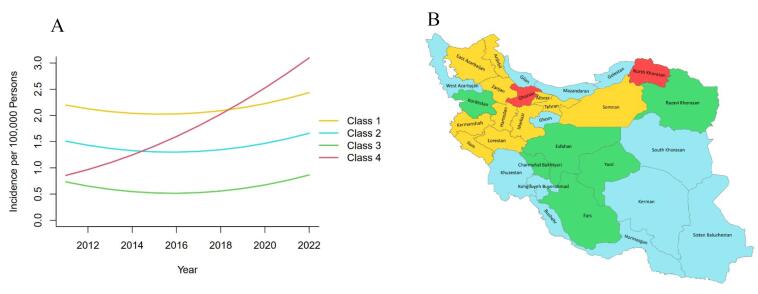
Male Carbon Monoxide Poisoning Mortality Rates Across Time in a 5-Cluster Growth Mixture Model (Panel A), Geographic Illustration of GMM Classification for Trends in Different Classes from 2011 to 2022 (Panel B)

 For females, the GMM identified four distinct trajectories of COP mortality from 2011 to 2022. Class 1 provinces had the highest baseline rates and remained relatively stable. Class 2 provinces, starting from the lowest levels, showed the sharpest increase throughout the study period. Class 3 provinces began at moderate levels and exhibited gradual improvement, while Class 4 provinces followed an intermediate path with a steady upward trend (Figure 3, Table S3, Table S4). For males, four distinct patterns were also observed. Class 1 provinces showed the highest initial mortality rates with only slight annual increases. Class 2 provinces started from low levels but experienced rapid acceleration, representing the strongest upward trend. Class 3 provinces, including large regions, began with higher mortality but gradually declined. Class 4 provinces showed moderate baseline levels with consistent upward increases until 2022 (Figure 4, Table S3, Table S4).

**Figure 5:**
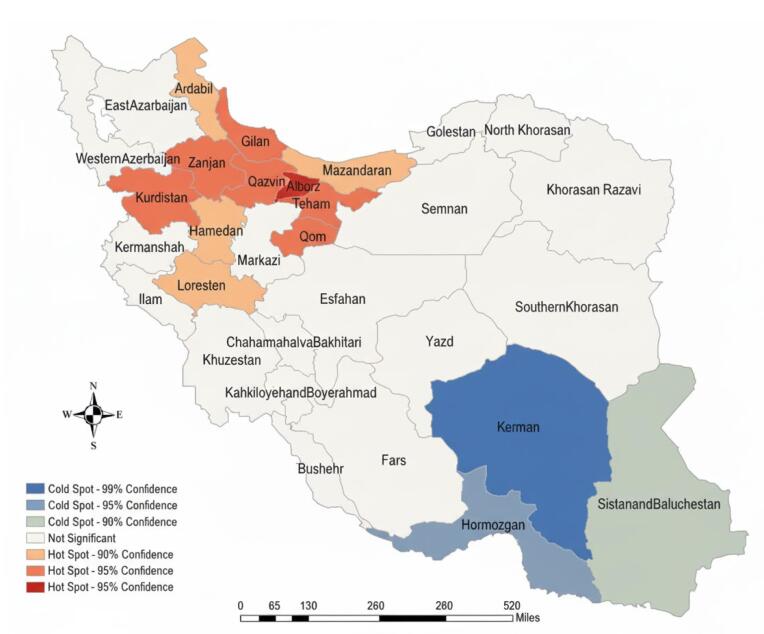
Hotspots and Coldspots of Average Carbon Monoxide Poisoning (COP) Mortality Rate in Iran by Province, 2011–2022. Clusters were identified using the Getis-Ord Gi* statistic (|Z| > 1.96, *P* < 0.05). Hotspots are red, coldspots are blue, and non-significant areas are gray. Rates are per 100,000 population

 The Global Moran’s I statistic indicates significant spatial autocorrelation in COP mortality rates across the 31 provinces (Moran’s I = 0.139, Z = 2.008, *P* = 0.044), indicating a non-random spatial pattern. Consistent with this, the Getis-Ord Gi spatial analysis revealed distinct geographic clustering at different confidence thresholds. At the 95% confidence level, a contiguous high-risk zone emerged across northwestern and central Iran, encompassing the Gilan, Zanjan, Qazvin, Tehran, Qom, and Kurdistan provinces, where elevated mortality rates demonstrated strong spatial dependence. Simultaneously, the Hormozgan province in southern Iran stood out as a statistically significant coldspot with consistently low mortality rates. Extending the analysis to the more stringent 99% confidence level (|Z| > 2.58) revealed additional critical patterns. Alborz emerged as a high-intensity hotspot, exhibiting exceptionally elevated mortality rates with robust statistical significance. In contrast, Kerman was identified as a pronounced coldspot with remarkably low mortality rates. These findings demonstrate a clear spatial polarization of COP mortality risk, with the most statistically significant hotspots concentrated in northwestern and central regions and the most definitive coldspots located in southeastern provinces (Figure 5, Figure S2).

## Discussion

 The results of this study offer significant insights into the mortality rates of COP in Iran from 2011 to 2022. Despite considering noticeable heterogeneity across the Iranian provinces, the overall crude linear mortality rate trend of COP tends to be stable with few fluctuations. Our quadratic GMM analysis revealed a J-shaped trend at the national level. This trend was more pronounced among males than females. After clustering provinces using GMM, the results indicated that the mortality rate in classes 2 and 3 increased by 3 and 2 times during 12 years, respectively, while a decreasing trend was observed in class 5. A previous study in Iran, regardless of the high variation between provinces, reported a decreasing mortality trend in Iran.^7^

 In the present study, an increasing trend in mortality due to COP was observed, which is inconsistent with the study by Alimohamadi et al and other studies conducted in Iran.^7,10,11^. The inconsistency between our findings of an increasing trend in COP mortality and the previous study’s reported decrease^7^ can be attributed to two critical factors: methodological differences in modeling temporal trends and extended time coverage. The prior study relied on linear trend analysis (visualized through line plots), which assumes a constant rate of change over time. While this approach suggested an overall decline from 2011 to 2018, it inherently masked potential non-linear patterns, such as inflection points or late-stage resurgences. In contrast, using GMM explicitly accounts for non-linear trajectories by incorporating quadratic terms, enabling detection of complex trends like the J-shaped curve observed nationally. This model revealed an initial decline (2011–2016) followed by a sharp upward turn (2017–2022), a pattern invisible to linear methods. Furthermore, the previous study’s endpoint in 2018 coincided with the trough of the J-curve, creating an illusion of sustained decline. Extending the analysis to 2022, we captured the post-2018 resurgence in mortality rates, which reversed earlier gains. Thus, the linear method’s inability to detect non-linearity, combined with truncated time coverage, led to an incomplete trend interpretation. Our findings underscore the necessity of advanced modeling techniques like GMM to avoid misleading conclusions in public health surveillance.

 Our study indicated that the annual COP mortality rate in Iran increased from 2011 to 2022, with approximately 0.017 per 100,000 people per year. Although the global trend of COP mortality rates has decreased by 36% over the past two decades, the situation in Iran is quite different.^8,9^ Compared to other countries, the United States and Canada have reported an annual reduction in mortality rates of approximately 5% over the last 30 years.^26,27^ Similar studies conducted in European countries such as Greece, France, Spain, and the United Kingdom reported a downward trend in mortality rates.^28-30^ However, a few countries, like Central Sub-Saharan Africa and Eastern Sub-Saharan Africa, have shown an increasing trend.^8^ Although the COP death rate in Turkey, as a cold region in western Iran, remained stable from 2008 to 2017.^31^ Regarding variation between countries, studies have shown that economic recessions in some regions lead to outdated or unsafe heating methods, which can increase the risk of COP.^8,32^ Additionally, low-income households may not have access to modern and safe heating tools and may use older appliances that are more prone to CO leakage. These households may also be unable to afford CO detectors or regular maintenance for their heating systems, further increasing their vulnerability to poisoning.^3,12^ Likewise, global and regional studies indicated that the sharpest overall decreases were observed in countries with high SDI, like Estonia, South Korea, and Puerto Rico.^8^

 Across the cold, mountainous provinces of Alborz, East Azerbaijan, Zanjan, North Khorasan, Qazvin, and Semnan (Class 1), persistently high yet flat CO-poisoning mortality may reflect heavy reliance on natural-gas heaters that often go unmaintained, allowing chronic low-level CO leaks to continue undetected throughout the winter months.^33,34^ Although more than 95% of urban households nationwide are connected to piped natural gas, irregular maintenance of gas appliances and chimney systems likely sustains a steady background exposure rather than episodic spikes in fatalities.^33,34^ At the same time, proximity to major referral centers in Tabriz and Tehran may facilitate timely diagnosis and treatment of CO poisoning, buffering against sudden surges in mortality.^35^

 In Ardabil, Ilam, and Markazi (Class 2), where mortality rates have nearly tripled from 0.72 to 2.15 per 100,000, rapid urban and industrial expansion may have increased the use of gas-fired space heaters and breakdown-prone gasoline generators during frequent power outages, compounding indoor CO accumulation.^34,36^ Public awareness appears low (fewer than 2% of homes in smaller cities install CO detectors), so many residents may remain unaware of creeping CO levels until severe symptoms develop.^33^ Prolonged, harsh winters in these highland regions may further extend the heating season, potentially amplifying cumulative CO exposure over successive years.^34^

 By contrast, coastal and southern provinces such as Bushehr, Hormozgan, and Sistan & Baluchestan (Class 3) exhibit low, stable mortality, possibly because milder climates reduce reliance on indoor combustion heating, and mixed-fuel use (e.g. LPG, electricity) dilutes dependence on natural-gas appliances. Nonetheless, under-reporting in dispersed rural communities (where emergency services and vital registration systems are less accessible) may conceal the actual burden of CO-related deaths.^7^

 Mazandaran and South Khorasan (Class 4) have shown moderate upward trends that may reflect their recent extension of piped gas to rural areas without concomitant safety inspection programs, leaving new users without guidance on appliance installation or maintenance.^33^ The combination of humid-cold winters in Mazandaran and chilly desert nights in South Khorasan prompts intermittent heater use in poorly ventilated rooms, while public health outreach on CO safety remains patchy in remote districts.^37^

 Finally, the eleven provinces in Class 5 (including Tehran, Esfahan, Fars, Chaharmahal & Bakhtiari, Hamedan, Kohgiluyeh & Boyerahmad, Kermanshah, Razavi Khorasan, Kurdistan, Lorestan, Qom, and Yazd) started with high mortality but now show gradual declines, likely owing to mature urban gas networks with regular safety inspections, subsidized CO-alarm distribution, and extensive media campaigns that have incrementally raised household vigilance.^33,35^ Access to healthcare services, including ambulance coverage in these major centers, further ensures rapid emergency response and effective treatment of poisoning cases.^38^ However, the results showed that some of the western and northern provinces, such as Kurdistan and Ardabil, had an increasing trend in deaths from CO poisoning, which is consistent with previous studies.^5,7^ Various studies in northwestern Iran, including Ardabil, Tabriz, and other regions, have shown that this pattern could be due to the cold weather in these regions, the widespread use of gas heaters, high dependence on natural gas as the main energy source, the expansion of the gas supply network, and lack of sufficient awareness regarding the safety and proper installation of gas appliances.^36,39,40^

 To address these divergent trends, tailored interventions are needed. In Class 1, provincial governments could subsidize annual professional servicing of domestic gas appliances and chimneys, especially before winter, and deploy mobile maintenance units to reach remote villages. Class 2 regions would benefit from targeted awareness campaigns via local media and community centers, voucher-based CO-detector distribution, and stricter regulations on generator room ventilation. In Class 3, strengthening rural death registration systems by training community health workers to recognize and report CO-related symptoms, alongside equipping peripheral clinics with portable CO-oximeters, could improve surveillance and prevention. For Class 4, gas companies and local councils might offer free “starter pack” inspections when new connections are made, including practical demonstrations of safe installation and ventilation procedures. Finally, in Class 5, integrating real-time odorant-monitoring sensors into urban gas networks and maintaining public reminders to service aging appliances would help sustain and accelerate mortality declines nationwide.

 In the current study, the male-to-female ratio was 2.87, confirmed by global studies.^8,9,26,28,29,31,32,41^ Also, this result is consistent with a systematic review in Iran.^5^ The higher exposure of men to carbon monoxide (CO) is attributed to several factors, including their greater use of CO-emitting devices, increased utilization of fuel-related equipment, and higher engagement in occupations where the risk of CO exposure is more prevalent compared to women.^27,36^ Furthermore, the COP mortality rates in the northern and western provinces were higher. One of the reasons is the cold weather in these areas. Studies conducted in western, northern, and northwestern Iran have also shown that in these areas, COP occurs more frequently in the cold seasons.^35,39,40,42^ The study by Dianat and Nazari in northwestern Iran showed that one of the main reasons for the high number of deaths in this region was lack of knowledge about the correct installation of heating equipment and the incorrect use of gas appliances.^40^ Another study in northwestern Iran indicated that the poor safety information of people about heating appliances is one of the predisposing factors of COP.^36^ Studies have also shown that COP occurs more frequently in cold regions and cold seasons, like February and March.^27-29,31^

 Limitations of this study include the unavailability of individual data, which could lead to ecological fallacy if one infers individual-level risk from aggregated data. In addition, disaggregation of deaths by incident location, such as home or workplace, was also unavailable. It is recommended that future studies investigate the trend of carbon monoxide deaths based on individual data and the location of the incident. Another limitation of this study is the possibility of misclassification of exposure or undercounting/misclassification of CO-related deaths in this dataset, especially in remote villages. In these areas, administrative processes, including the reporting and investigation of suspicious deaths due to homicide, suicide, accidents, and other causes, may not be conducted by medical examiners. Instead, burials may occur with only local testimony from villagers, without issuing a burial permit or a precise determination of the cause of death. To address this issue, establishing an online registration system to document deaths based on evidence and clues in these areas, as well as developing a more accurate and sensitive registration system to detect and report deaths from COP in the country, could help reduce underreporting. Another limitation of this study is that the models used lack key exposure-related variables such as altitude, temperature, poverty, or living environment ventilation conditions that may play an important role in analyzing trends and regional differences. It is recommended that future studies examine these variables and include them in their models. The strength of the present study is that previous studies have reported a 7-year trend. In contrast, the present study examined a 12-year trend. In addition, our study using GMM analysis showed that the mortality rate from COP in Iran has high heterogeneity and that the mortality rate of most provinces has increased significantly over the past decade.

## Conclusion

 This study found that CO-poisoning mortality in Iran rose overall between 2011 and 2022, with nearly half of the provinces experiencing substantial increases and apparent regional disparities in trend and spatial clustering. Notably, northwestern and central provinces (identified as hotspots) should be prioritized for intervention. Provinces with persistently high or rapidly rising rates highlight the need for area-focused measures to enhance appliance safety and broaden access to modern, well-maintained heating systems, particularly in low-income households most at risk. Equally important is raising public awareness about safe heater use and promoting CO-detector installation, especially in colder regions where exposure peaks. By aligning these broad, targeted actions with local needs, Iran can meaningfully reduce its CO-poisoning burden and improve population health.

## Supplementary Files

Supplementary file 1 contains Tables S1-S4 and Figures S1-S2.
