# Correction to: Latent Trajectories and Regional Differences in Carbon Monoxide Mortality Across Provinces of Iran

**DOI:** 10.34172/aim.35526

**Published:** 2026-01-01

**Authors:** Farzad Maleki, Zahedeh Khoshnazar, Azadeh Noohi, Mohammad Reza Taherian, Fatemeh Majdolashrafi, Seyed Saeed Hashemi Nazari

**Affiliations:** ^1^Department of Epidemiology, School of Public Health & Safety, Shahid Beheshti University of Medical Sciences, Tehran, Iran; ^2^Air Quality and Climate Change Research Center, Research Institute for Health Sciences and Environment, Shahid Beheshti University of Medical Sciences, Tehran

 In the article titled *“Latent Trajectories and Regional Differences in Carbon Monoxide Mortality Across Provinces of Iran,”* published in *Arch Iran Med. August 2025;28(8):432-442(doi:10.34172/aim.34565)*, the affiliation of Seyed Saeed Hashemi Nazari should be updated to the following:

**Air Quality and Climate Change Research Center, Research Institute for Health Sciences and Environment, Shahid Beheshti University of Medical Sciences, Tehran.**

 This correction has now been updated in both the PDF and HTML versions of the article.

