# Supplementary material for: Latent Trajectories and Regional Differences in Carbon Monoxide Mortality Across Provinces of Iran
Source: Arch Iran Med. 2025 Aug 1;28(8):432–42. doi: 10.34172/aim.34565 (PMC12569986; doi:10.34172/aim.34565)
Supplement: Supplementary file 1 — contains Tables S1-S4 and Figures S1-S2. [file aim-28-432-s001.pdf]

Arch Iran Med. August 2025;28(8):432-S1
10.34172/aim.34565
http://journalaim.com

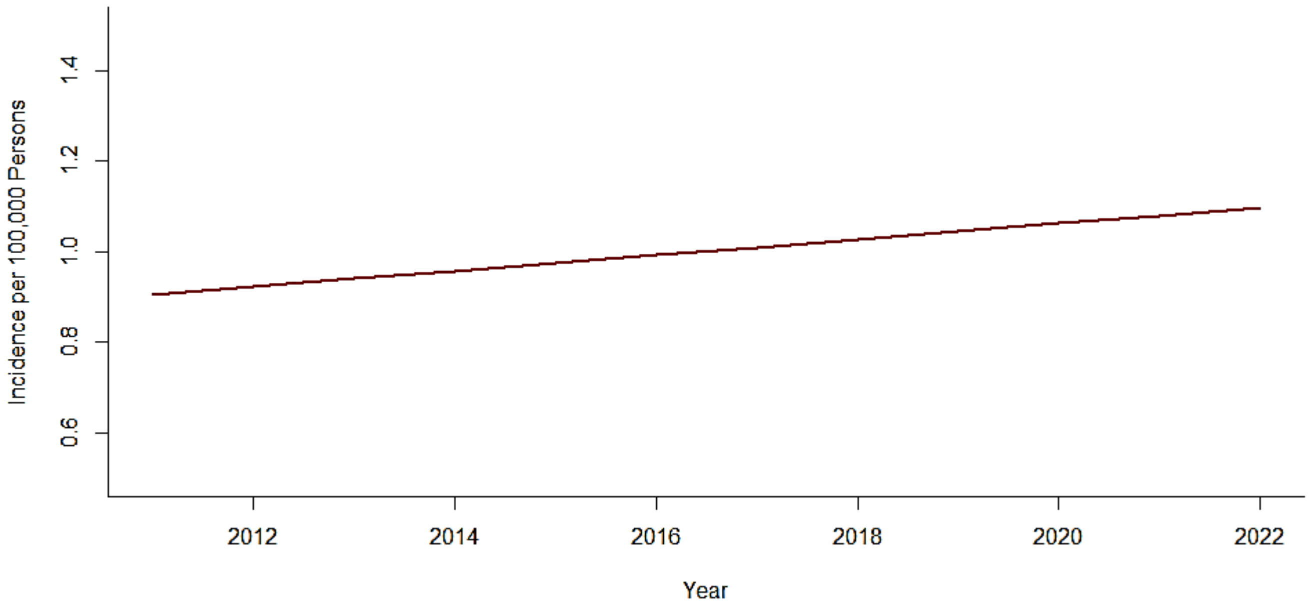

Figure S1: Overall linear trend of COP mortality rate using GCM in Iran, 2011-2022.

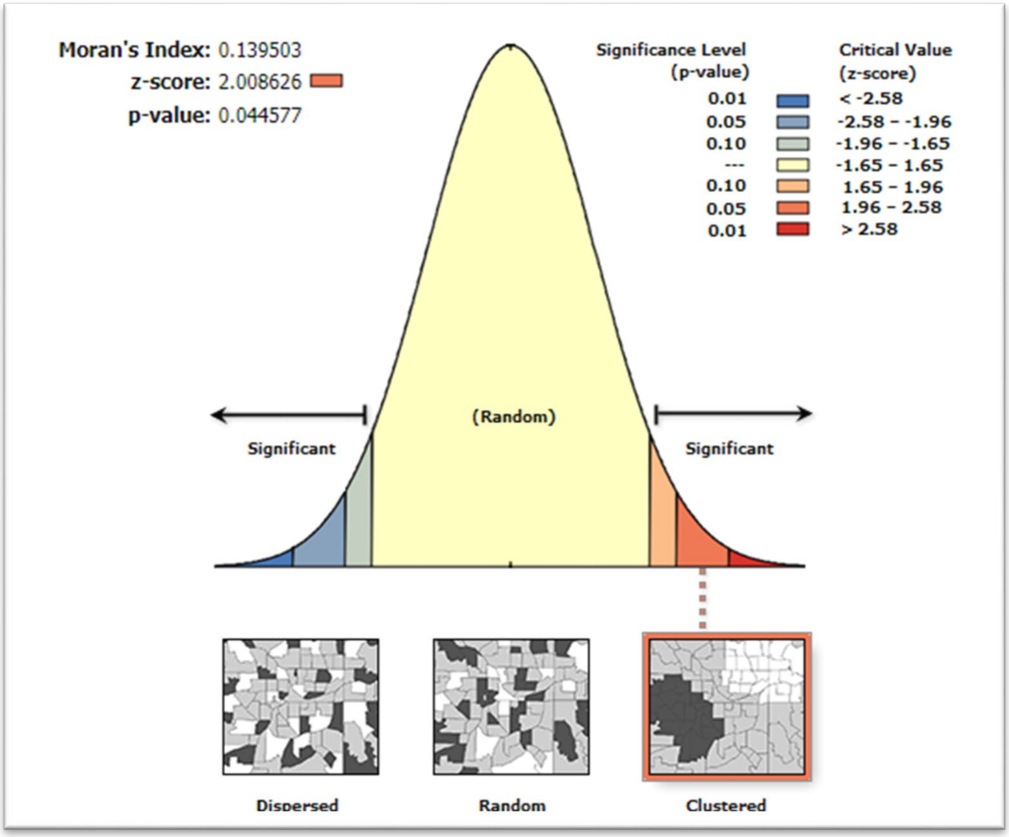

Figure S2. Z-score of Moran's autocorrelation has a similar and clustered pattern (z-score: 2.01, p-value: 0.04).

Table S1. Growth Mixture Models Results for Carbon Monoxide Mortality Rate in Iran, 2011-2022.

Model	Term	Coefficient	Standard Error	Wald Statistic	p-value
Overall	Intercept	1.106	0.109	10.171	<0.0001
	Slope	-0.076	0.023	-2.853	<0.004
	Quadratic Term	0.007	0.002	-2.85	<0.001
Male	Intercept	1.521	0.159	9.526	<0.0001
	Slope	-0.086	0.042	-2.046	<0.041
	Quadratic Term	0.009	0.003	2.922	<0.003
Female	Intercept	0.684	0.078	8.677	<0.0001
	Slope	-0.066	0.021	-3.234	<0.001
	Quadratic Term	0.0052	0.0015	3.339	<0.008

Table S2: Fitness indices of estimated Latent Growth Mixture Models with Different Numbers of Classes of Carbon Monoxide Death Rates (Incidence/100,000) in both sexes in 31 Provinces in Iran, 2011–2022.

Sex	Fit Indices	Number of Classes						
		1	2	3	4	5	6	7
Male	AIC	813.29	811.45	810.85	807.13	814.57	821.57	805.07
	BIC	820.46	826.35	844.68	825.77	835.23	857.42	858.65
	ssBIC	804.88	786.31	785.27	779.25	782.26	779.53	778.31
	Entropy	1	0.891	0.905	0.918	0.914	0.899	0.811
Female	AIC	295.16	282.04	283.88	279.95	287.78	296.38	302.34
	BIC	315.64	300.67	308.26	292.86	317.9	332.23	343.92
	ssBIC	271.81	264.82	260.18	252.47	255.29	254.34	253.57
	Entropy	1	0.844	0.678	0.867	0.847	0.784	0.766
Overall	AIC	476.42	473.61	463.19	468.21	459.61	480.64	487.31
	BIC	483.59	503.72	481.83	492.58	472.51	516.49	528.89
	ssBIC	468.01	438.3	441.33	439.62	444.47	438.6	438.54
	Entropy	1	0.901	0.924	0.843	0.937	0.881	0.876

AIC Akaike information criterion, BIC Bayesian information criterion, ssBIC Sample-Size adjusted BIC

Table S3: Estimates of Growth Mixture Model Parameters for carbon monoxide death rate (Incidence per 100,000) in both sexes in 31 provinces, Iran, 2011–2022.

Sex	Class	Mean* 2011	Mean* 2022	Intercept		Slope		Quadratic Term
				Estimate	SE	Estimate	SE	
Male	1	2.19	2.44	2.29	0.18	0.05	0.03	0.007
	2	1.52	1.66	1.61	0.16	0.01	0.01	0.007
	3	0.71	0.85	0.76	0.26	0.48	0.24	0.007
	4	0.84	3.12	0.83	0.18	0.53	0.18	0.007
Female	1	1.39	0.77	1.49	0.18	-0.05	0.02	0.005
	2	0.69	0.43	0.77	0.11	-0.02	0.01	0.005
	3	0.29	0.38	0.34	0.08	0.01	0.01	0.005
	4	0.82	1.04	0.86	0.09	0.02	0.01	0.005
Overall	1	1.71	1.86	1.78	0.12	0.01	0.01	0.005
	2	0.72	2.15	0.68	0.17	0.13	0.02	0.006
	3	0.51	0.59	0.59	0.12	0.01	0.01	0.006
	4	0.68	1.18	0.72	0.29	0.04	0.04	0.006
	5	1.25	1.14	1.34	0.12	-0.01	0.01	0.006

*Mean per 100,000 population based on the GMM model.

Table S4: Distribution of provinces by carbon monoxide death Rate (Incidence/100,000) in Both Genders in 31 provinces of Iran, 2011–2022.

Sex	Class	N (%)	Provinces
Male	Class 1	11 (35)	Fars, Hamedan, Kohkiloyeh and Boyerahmad, Kermanshah, Khorasan Razavi, Kurdistan, Lorestan, Mazandaran, Qom, Southern Khorasan, Yazd
	Class 2	9 (29)	Alborz, Charmahal and Bakhtiari, East Azarbaijan, Esfahan, North Khorasan, Qazvin, Semnan, Tehran, Zanjan
	Class 3	8 (26)	Bushehr, Gilan, Golestan, Hormozgan, Kerman, Khuzestan, Sistan and Baluchestan, West Azerbaijan
	Class 4	3 (10)	Ardabil, Ilam, Markazi
Female	Class 1	11 35)	Alborz, Ardabil, East Azarbaijan, Hamedan, Ilam, Kermanshah, Lorestan, Markazi, Semnan, Tehran, Zanjan
	Class 2	12 39)	Bushehr, Gilan, Golestan, Hormozgan, Kohkiloyeh and Boyerahmad, Kerman, Khuzestan, Mazandaran, Qom, Sistan and Baluchestan, Southern Khorasan, Western Azerbaijan
	Class 3	6 (31)	Charmahal and Bakhtiari, Esfahan, Fars, Khorasan Razavi, Kurdistan, Yazd
	Class 4	2 (6)	North Khorasan, Qazvin
Overall	Class 1	6 (19)	Alborz, East Azarbaijan, North Khorasan, Qazvin, Semnan, Zanjan
	Class 2	3 (10)	Ardabil, Ilam, Markazi
	Class 3	8 (26)	Bushehr, Gilan, Golestan, Hormozgan, Kerman, Khuzestan, Sistan and Baluchestan, Wes Azerbaijan
	Class 4	2 (6)	Mazandaran, South Khorasan
	Class 5	12 39)	Charmahal and Bakhtiari, Esfahan, Fars, Hamedan, Kohkiloyeh and Boyerahmad, Kermanshah, Khorasan Razavi, Kurdistan, Lorestan, Qom, Tehran, Yazd
